# Indomethacin Treatment of Mice with Premalignant Oral Lesions Sustains Cytokine Production and Slows Progression to Cancer

**DOI:** 10.3389/fimmu.2016.00379

**Published:** 2016-09-22

**Authors:** Sara D. Johnson, M. Rita I. Young

**Affiliations:** ^1^Research Service, Ralph H. Johnson VA Medical Center, Charleston, SC, USA; ^2^Department of Pathology and Laboratory Medicine, Medical University of South Carolina, Charleston, SC, USA; ^3^Department of Otolaryngology – Head and Neck Surgery, Medical University of South Carolina, Charleston, SC, USA

**Keywords:** head and neck cancer, HNSCC, immune, premalignant oral lesions, cytokines, T cell

## Abstract

Current treatment options for head and neck squamous cell carcinoma (HNSCC) patients are often ineffective due to tumor-localized and systemic immunosuppression. Using the 4-NQO mouse model of oral carcinogenesis, this study showed that premalignant oral lesion cells produce higher levels of the immune modulator, PGE_2_, compared to HNSCC cells. Inhibiting prostaglandin production of premalignant lesion cells with the pan-cyclooxygenase inhibitor indomethacin stimulated their induction of spleen cell cytokine production. In contrast, inhibiting HNSCC prostaglandin production did not stimulate their induction of spleen cell cytokine production. Treatment of mice bearing premalignant oral lesions with indomethacin slowed progression of premalignant oral lesions to HNSCC. Flow cytometric analysis of T cells in the regional lymph nodes of lesion-bearing mice receiving indomethacin treatment showed an increase in lymph node cellularity and in the absolute number of CD8^+^ T cells expressing IFN-γ compared to levels in lesion-bearing mice receiving diluent control treatment. The cytokine-stimulatory effect of indomethacin treatment was not localized to regional lymph nodes but was also seen in the spleen of mice with premalignant oral lesions. Together, these data suggest that inhibiting prostaglandin production at the premalignant lesion stage boosts immune capability and improves clinical outcomes.

## Introduction

The 5-year survival rate for patients with head and neck squamous cell carcinoma (HNSCC) remains at ~50% (1). Traditional treatments for HNSCC patients, including surgical resection and chemo-radiation, often fail due to the high incidence of cancer recurrence. There exists a critical need for more effective therapies, and there has been a recent interest in incorporating immunotherapies in the treatment plan for patients with HNSCC. However, the effectiveness of immunotherapy may be compromised by the pronounced immune inhibition in HNSCC patients (2–4). Established HNSCC tumors use a variety of mechanisms to thwart the T cell-mediated antitumor response, including downregulation of MHC-II molecule expression, upregulation of death ligand expression (FasL) and direct secretion of several immunosuppressive molecules, including TGF-β and PGE_2_ (5, 6).

The role of PGE_2_ in the tumor environment is multifold. PGE_2_ has been shown to promote tumor cell growth and invasiveness and to also contribute to tumor angiogenesis (7–10). Increased levels of tumor-localized cyclooxygenase-2 (COX-2), the enzyme responsible for increased PGE_2_ production, are associated with increased levels of immunosuppressive cells at the tumor site and a worse prognosis in patients with oral cancer (4, 11–14). PGE_2_ has been shown to directly inhibit T cell proliferation and IFN-γ production, and to skew responses toward a Th2 phenotype (15). Tumor-derived PGE_2_ has also been shown to chemoattract immune inhibitory cells, such as MDSC (16). In some types of malignancies, PGE_2_ has been shown to induce immune cell production of IL-10 and directly suppress the production of proinflammatory cytokines by CD4^+^ T cells (4, 15, 17). In several murine and hamster models of head and neck cancer, administration of the COX-2 inhibitor celecoxib decreased expression of vascular endothelial growth factor (VEGF) and survivin, increased tumor cell apoptosis and decreased tumor growth (18–20). A few studies have shown clinical responses resulting from COX inhibition in HNSCC patients (21, 22). In a model of lung carcinoma, inhibition of COX-2 resulted in increased antitumor reactivity by shifting the cytokine balance of resident immune cells toward a more proinflammatory, Th1-like phenotype, as characterized by increased IL-12 production and decreased IL-10 production (23). While, the immune modulating role of PGE_2_ in the tumor environment has been studied, its role in immune modulation as premalignant oral lesions progress to HNSCC tumors has not been previously investigated.

Using the 4-NQO mouse model of oral carcinogenesis, our prior studies had demonstrated significant immune changes at the premalignant lesion stage, before HNSCC tumors are established (24). The 4-NQO mouse model is based on the carcinogenic effects of 4-NQO, which mimics the effects of tobacco at the molecular level and produces all stages of oral carcinogenesis (25, 26). Cervical lymph nodes of mice with 4-NQO induced premalignant lesions have been characterized to have increased levels of stimulatory/inflammatory cells and cytokines, compared to lymph nodes of both control mice and HNSCC-bearing mice (24). In contrast, tumor-draining lymph nodes of HNSCC-bearing mice contain increased levels of immunosuppressive Foxp3^+^ T regulatory cells and functionally compromised conventional T cells. These studies suggest that an activated T cell response is occurring at the premalignant lesion stage that wanes as the immunosuppressive environment of HNSCC is established. The premalignant lesion cells contribute to the inflammatory phenotype by their production of G-CSF, RANTES, MCP-1, and PGE_2_ (27). Because of the progressive nature of this model, which spans from premalignant oral lesions to HNSCC, investigation into the immune environment during the course of progression is possible.

The current study aimed to investigate the effect of inhibiting PGE_2_ production in mice with 4-NQO-induced premalignant oral lesions on tumor progression and T cell reactivity. These studies showed that administration of indomethacin, a pan-COX inhibitor, to mice with premalignant oral lesions increased immune cell cytokine production and improved clinical outcomes.

## Materials and Methods

### Oral Carcinogenesis Model

Two-month-old female C57BL/6 mice (Charles Rivers Laboratory, Wilmington, MA, USA) were administered 4-NQO at a final concentration of 50 μg/ml in their drinking water until the development of premalignant oral lesions. Premalignant oral lesions typically appear on the tongue at 6–8 weeks of 4-NQO treatment and gradually progress to HNSCC. To monitor lesion development, mice were examined endoscopically weekly using a Stryker 1.9 mm × 30°mm endoscope and images were taken using a Stryker 1088 camera (Kalamazoo, MI, USA). During the procedure, mice were sedated with inhaled isoflurane (Piramal Healthcare, Boise, ID, USA). All animal procedures were conducted under the approval of the Institutional Animal Care and Use Committee (IACUC) of the Ralph H. Johnson VA Medical Center. All study procedures were in compliance with institutional and federal guidelines regulating the care and use of laboratory animals in research.

### Culture Medium

Cell culture media consisted of 1× DMEM (Life Technologies, Grand Island, NY, USA) containing 4.5 g/l d-glucose and l-glutamine, supplemented with 10% fetal bovine serum (FBS) and 1× antibiotic antimycotic solution, containing penicillin, streptomycin, and amphotericin B (Sigma, St. Louis, MO, USA). To establish cell lines from premalignant lesions and HNSCC tumors, culture media was supplemented with 2× antibiotic antimycotic solution for the first 2 weeks of culture.

### Premalignant Lesion and HNSCC Primary Cell Lines

Primary cell lines were established, as previously described (27) by excising 4-NQO-induced premalignant lesions or HNSCC from the tongues of premalignant lesion- or HNSCC-bearing mice at the appropriate stage, as defined through histopathological analysis by the oral pathology section in the Center for Oral Health Research at the Medical University of South Carolina. Briefly, excised premalignant lesions or HNSCC tumors were placed into culture and, once adherent cells became established, non-adherent cells were removed. Prior to defining cells as premalignant or HNSCC, their epithelial phenotype was confirmed, as well as uniformity of their microscopic and growth characteristics. Supernatants were collected from sub-confluent cultures (80%) after 48 h and used in the specific analyses described below.

### Spleen Cell Preparation

Spleens were harvested from healthy control C57BL/6 mice and homogenized using a glass homogenizer. Cells were passed through a 70 μm cell strainer (BD Falcon, San Jose, CA, USA) and rinsed with HBSS (Life Technologies, Grand Island, NY, USA). Red blood cells were lysed by adding ACK Lysing Buffer (Lonza, Walkersville, MD, USA) for 3 min. Splenocytes were then washed twice with HBSS. Cell number was determined by counting cells excluding trypan blue using a hemocytometer.

### Spleen Cell Culture with Supernatants of Indomethacin-Treated Premalignant Lesion Cells or HNSCC Cells

Premalignant lesion cells and HNSCC cells were cultured for 24 h at 37°C at 1 × 10^6^ cells/well in 12-well tissue culture plates in fresh media with or without 6 μg/ml indomethacin, as previously described (28). Supernatants were then collected for culture with spleen cells. Spleen cells from healthy control C57BL/6 mice were cultured for 72 h at 37°C with diluted (1:2 in fresh medium) supernatants collected from indomethacin- or diluent control-treated premalignant lesion cells and HNSCC cells at 1 × 10^6^ cells/well in 12-well anti-CD3-coated tissue culture plates with 30 IU mouse IL-2. Controls consisted of splenocytes cultured for 72 h at 37°C in fresh medium, with or without 6 μg/ml indomethacin. Supernatants were collected from the spleen cell cultures and the levels of cytokines were quantitated. Also quantitated were levels of cytokines or PGE_2_ in the media conditioned by premalignant lesion cells or HNSCC prior to its addition to the spleen cells.

### Quantitation of Secreted Cytokines by Cytokine Bead Array

All reagents used for the cytokine bead array were from BD Biosciences (San Jose, CA, USA). The levels of IFN-γ, IL-2, IL-17A, IL-6, TNF-α, and IL-10 in cell culture supernatants were determined using a mouse cytometric bead array Th1/Th2/Th17 cytokine kit. A FACS Canto (BD Biosciences) flow cytometer was used to quantify cytokine profiles and relative amounts of each cytokine were analyzed using the FCAP Array Software (manufactured by Soft Flow Hungary Ltd. for BD Biosciences). Cytokine levels in premalignant lesion cell and HNSCC cell supernatants were measured before addition to cultures with spleen cells, and these levels were subtracted from the total cytokine levels in the supernatants from spleen cells that were co-cultured with lesion or HNSCC-conditioned media. Cytokine levels from a representative experiment are shown in pg/ml. To standardize cytokine levels produced by spleen cells cultured in diluent- or indomethacin-containing fresh medium, data from five independent experiments run in duplicate are presented as fold change/control.

### Indomethacin Treatment of Premalignant Lesion-Bearing Mice

Two-month-old female C57BL/6 mice were administered 4-NQO in their drinking water until the development of premalignant oral lesions (8 weeks), as determined by weekly endoscopic examination of the oral cavities. At this point, 4-NQO water was removed and replaced with drinking water containing either 30 μg/ml indomethacin (10 mice) or diluent (1% ethanol) control (10 mice) (29). Oral cavities were endoscopically monitored every 10 days using a Stryker 1.9 mm × 30°mm endoscope and lesions were counted and scored blindly. Lesion progression was documented using a Stryker 1088 camera. At 6 and 20 weeks after beginning indomethacin or diluent control treatment, five mice per group were sacrificed and cervical lymph nodes were harvested. A group of age-matched untreated C57BL/6 healthy control mice were also sacrificed at these time points for collection of cervical lymph nodes.

### Lesion Scoring

As part of the endoscopic examination performed every 10 days, the oral cavity was photographed. Using the photographic images, the total number and gross pathologic score of tongue lesions in 4-NQO-treated mice receiving either indomethacin or diluent control were blindly assessed, as previously described (24, 30). Lesions were scored on a 1–4 scale, with 1 indicating a flat macule, 2 indicating a raised papule, 3 indicating a raised plaque, and 4 indicating a grossly exophytic lesion.

### Cervical Lymph Node Cell Preparation and Culture

Cervical lymph nodes harvested from experimental and control C57BL/6 mice were homogenized to a single cell suspension using a Stomacher 80 homogenizer (Seward Laboratory Systems, Davie, FL, USA), set on high for 90 s. Cells were then passed through a 70 μm cell strainer (BD Falcon, San Jose, CA, USA) and rinsed with HBSS. Cell number was determined by counting cells, excluding trypan blue using a hemocytometer. Cells were cultured in 12-well anti-CD3-coated tissue culture plates at 1 × 10^6^ cells/well in fresh media for 72 h at 37°C. Supernatants were collected (500 μl) from each well for cytokine quantitation by cytokine bead array. Cells were then restimulated with 2 μl/ml Cell Stimulation Cocktail (PMA/ionomycin; eBioscience, San Diego, CA, USA) and 0.6 μl/ml GolgiStop (BD Biosciences) for the last 4–6 h of culture at 37°C. Cells were gently collected from tissue culture plates with 25 cm cell scrapers (Sarstedt, Inc., Newton, NC, USA), washed once with stain buffer, and transferred to polystyrene tubes at 1 × 10^6^ cells/tube for flow cytometric analysis.

### Flow Cytometric Analysis of Cells

All antibodies and reagents used for flow cytometric analysis were from BD Biosciences. Spleen cells or cervical lymph node cells were suspended in polystyrene tubes at 1 × 10^6^ cells/tube in 300 μl buffer, containing 2% FBS in sterile 1× PBS, at 4°C for 15 min and then centrifuged at 1,000 rpm for 5 min. Cells were resuspended in 10 μl Fc block, containing anti-CD16/CD32 antibody at a 1:100 dilution in sterile 1× PBS, at 4°C for 10 min to block non-specific antibody binding to the cell surface. After washing cells twice, they were resuspended in 1 ml cold BD Cytofix, and incubated for 20 min at 4°C. Cells were washed twice, resuspended in 50 μl BD Perm/wash buffer and incubated for 30 min at room temperature with equal concentrations of antibodies or appropriate isotype controls: FITC-CD4, APC-CD8a, and PerCP-Cy5.5-IFN-γ. After incubation with antibodies, cells were washed twice in BD Perm/wash buffer and resuspended in 400 μl BD stain buffer for analysis of the extent and frequency of positive-staining cells using a BD FACS Canto flow cytometer.

### Statistical Analyses

Data were reported using the mean as a measure of central tendency ± SEM. To compare one variable condition between groups, a one-way ANOVA analysis was initially performed (GraphPad Prism version 6.03 for Windows, GraphPad Software, La Jolla, CA, USA). If differences were identified by the ANOVA analysis, a two-tailed Student’s *t*-test or Mann–Whitney *U* test was then performed to determine significance of differences between each of two groups (e.g., control vs. premalignant, control vs. HNSCC, premalignant vs. HNSCC, and indomethacin vs. diluent control) using the GraphPad Prism version 6.03. Cohen’s *d* was used as the effect size measure for the Student’s *t-*test or Mann–Whitney *U* test. Significance was reported in the 95% confidence interval.

## Results

### Pretreatment of Premalignant Oral Lesion Cells with Indomethacin Skews Their Cytokine-Inducing Phenotype

**Figure 1:**
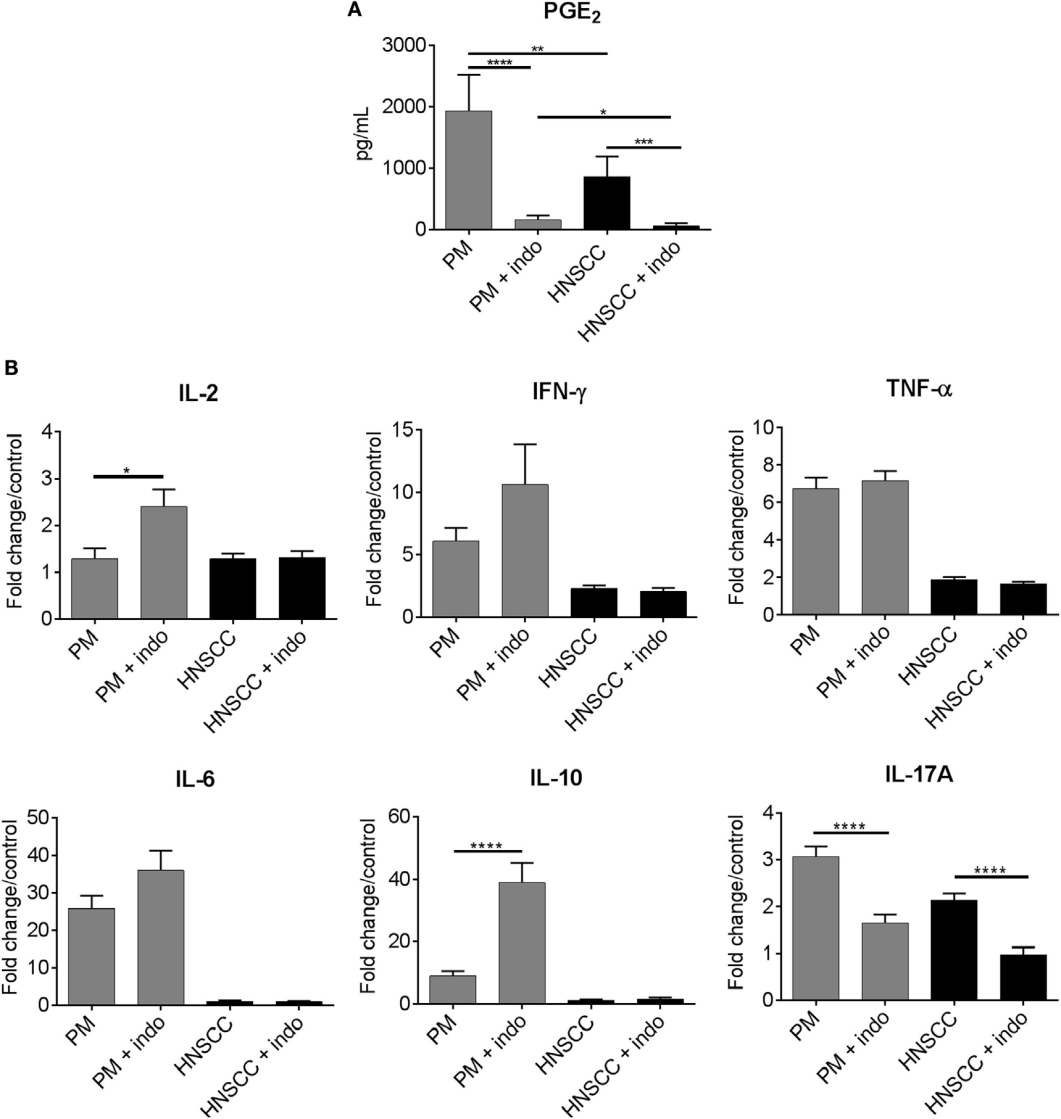
**Pretreatment of premalignant lesion cells with indomethacin skews their induction of spleen cell cytokine production toward increased production of Th1-associated cytokines and decreased production of IL-17A**. **(A)** Premalignant lesion cells (PM) and HNSCC cells were plated at 1 × 10^6^ cells/well and treated with 6 μg/ml indomethacin (indo) for 24 h. The levels of PGE_2_ in cell supernatants were measured by ELISA. **(B)** Spleen cells from healthy control C57BL/6 mice were cultured for 72 h with media conditioned by premalignant lesion cells or HNSCC cells that were treated with diluent or 6 μg/ml indomethacin. Supernatants were collected for cytokine measurement. Cytokine levels were standardized to levels produced by spleen cells cultured in diluent- or indomethacin-containing fresh medium instead of conditioned media and, thus, data are shown as fold change/control. Results are from five independent experiments, each run in duplicate. Data represent mean ± SEM. *p < 0.05, **p < 0.01, and ***p < 0.001 (two-tailed Student’s *t*-test).

One mechanism by which PGE_2_ impacts on immune reactivity in the tumor environment is by skewing immune cell cytokine production away from a Th1-type response (15, 17). To investigate how inhibiting PGE_2_ impacts on immune cell cytokine production in the premalignant oral lesion environment, spleen cells from healthy C57BL/6 mice were cultured with media conditioned by supernatants from indomethacin-treated premalignant lesion cells or HNSCC cells. The levels of cytokines produced by spleen cells (picogram per milliliter) are shown in Table 1. To more clearly represent the impact of indomethacin treatment on cytokine skewing by premalignant lesion cells and HNSCC cells, and to control for the impact of indomethacin on spleen cell cytokine production in control conditions, levels of cytokines in these cultures were normalized to the levels produced by spleen cells in the presence of equal concentrations of indomethacin in media alone (Figure 1).

**Table 1:** **Pretreatment of premalignant lesion cells with indomethacin skews their induction of spleen cell cytokine production**.

Cytokine (pg/ml)	Media	Media + indo	PM	PM + indo	HNSCC	HNSCC + indo
IL-2	181.5 ± 5.1	166.2 ± 4.1	258.5 ± 7.8	314.2 ± 5.6	178.9 ± 6.3	165.7 ± 2.8
	*p* = 0.0575		**p = 0.0012		*p* = 0.1069	
IFN-γ	94.8 ± 2.3	92.7 ± 1.5	483.2 ± 33.9	631.4 ± 20.2	139.5 ± 11.9	106.8 ± 2.9
	*p* = 0.4716		**p = 0.0095		*p = 0.0365	
TNF-α	45.5 ± 1.3	43.6 ± 1.1	495.9 ± 14.7	470.9 ± 8.7	67.9 ± 4.0	60.3 ± 2.7
	*p* = 0.3136		*p* = 0.1941		*p* = 0.1697	
IL-6	4.7 ± 0.7	3.2 ± 0.6	245.3 ± 8.9	243.8 ± 2.3	3.1 ± 1.1	3.4 ± 0.4
	*p* = 0.1477		*p* = 0.8718		*p* = 0.8034	
IL-10	2.6 ± 1.6	0.9 ± 0.9	52.0 ± 1.4	77.3 ± 1.1	6.3 ± 1.1	5.0 ± 1.7
	*p* = 0.3866		***p < 0.0001		*p* = 0.5423	
IL-17A	11.3 ± 0.7	10.8 ± 0.6	35.0 ± 0.4	17.0 ± 0.2	17.3 ± 0.4	6.4 ± 1.0
	*p* = 0.6005		***p < 0.0001		***p < 0.0001	

Spleen cells from healthy control C57BL/6 mice were cultured for 72 h with media conditioned by premalignant lesion cells or HNSCC cells that were treated with diluent or 6 μg/ml indomethacin. Supernatants were collected for cytokine measurement. Results are from a representative experiment performed in duplicate. Data are presented as picogram per milliliter and represent mean ± SEM. Significant values are indicated as follows: *p < 0.05, **p < 0.01, and ***p < 0.0001 (two-tailed Student’s *t*-test). PM, premalignant; HNSCC, head and neck squamous cell carcinoma; indo, indomethacin.

As shown in Table 1, media conditioned by premalignant lesion cells increased spleen cell production of the Th1 mediators IL-2 (*p* = 0.0002) and IFN-γ (*p* < 0.0001), the inflammatory mediators TNFα (*p* < 0.0001), IL-6 (*p* < 0.0001), and IL-17A (*p* < 0.0001), and the inhibitory mediator IL-10 (*p* < 0.0001) compared to media conditioned by HNSCC cells or media alone. Treatment of premalignant lesion cells and HNSCC cells with indomethacin significantly reduced their PGE_2_ production (Figure 1A). Indomethacin treatment of premalignant lesion cells further increased their induction of spleen cell production of Th1-associated cytokines IL-2 and IFN-γ (Figure 1B and Table 1). While media of indomethacin-treated premalignant lesion cells also increased spleen cell production of the inflammatory mediator IL-6, this difference was not statistically significant. Media conditioned by both premalignant lesion cells and HNSCC increased spleen cell production of IL–17A, but this stimulation was blocked, when premalignant lesion cells and HNSCC were treated with indomethacin. Interestingly, indomethacin treatment of premalignant lesion cells enhanced their stimulation of spleen cell production of IL-10, suggesting that inhibiting PGE_2_ production may also boost Th2-associated activity. With the exception of IL-17A, media conditioned by HNSCC cells did not impact on spleen cell production of any of the other cytokines, regardless of whether the HNSCC cells had been treated with diluent or with indomethacin. Overall, the data show that inhibiting prostaglandin production impacts on the premalignant lesion environment, boosting Th1- and Th2-associated cytokine secretion by immune cells.

### Administration of Indomethacin to Mice with 4-NQO-Induced Premalignant Lesions Improves Clinical Outcome

**Figure 2:**
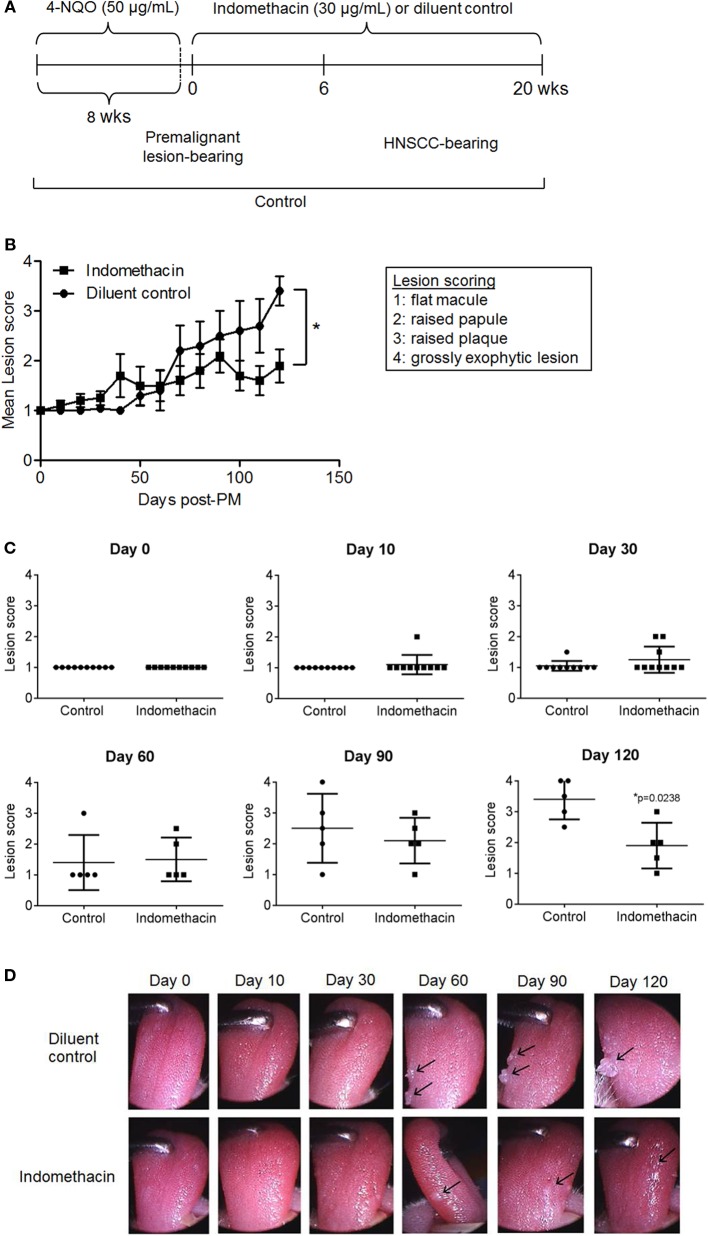
**Administering indomethacin to mice with 4-NQO-induced premalignant lesions slows progression to HNSCC**. **(A)** Indomethacin or diluent control was administered in the drinking water of mice with 4-NQO-induced premalignant oral lesions starting from when premalignant oral lesions were endoscopically detectable. **(B)** Endoscopic images of oral cavities were captured every 10 days and lesions were scored 1–4. **(C)** Lesion scores for each mouse at indicated time points. **(D)** Images of the oral cavity of a representative mouse from each group. Lesion score data represent mean ± SEM. Shown in **(C,D)** are lesion scores and endoscope images for mice scored at baseline and at days 13 (1.9 weeks), 33 (4.7 weeks), 70 (10 weeks), 104 (14.9 weeks), and 139 (19.9 weeks) after initiating indomethacin treatment. *p < 0.05 (Mann–Whitney *U* test).

Because inhibiting prostaglandin production by premalignant lesion cells resulted in their induction of increased cytokine production by immune cells, we sought to determine whether treating mice bearing 4-NQO-induced premalignant lesions with indomethacin to inhibit prostaglandin production would slow progression to tumor. For this study, 4-NQO was administered to 2-month-old C57BL/6 mice until the development of premalignant lesions, at which point the administration of indomethacin or diluent control was initiated and continued for 20 weeks (Figure 2A). To monitor the progression of premalignant lesions to HNSCC during the course of treatment, mice were examined by endoscopy every 10 days. The images captured of the oral cavity were stored and used for scoring the lesions in a double-blind manner. As shown in Figure 2B, mice that received indomethacin treatment from the time that their premalignant lesions were detectable had a significantly better overall clinical outcome compared to lesion-bearing mice receiving diluent control treatment. At the endpoint of the study (day 139, about 20 weeks post-initiation of indomethacin treatment), the average lesion score for mice receiving indomethacin was 1.9, whereas the average lesion score for mice receiving diluent control was 3.4. Differences in clinical outcome were more striking at later time points as opposed to earlier in the course of treatment. Although, the lower score for lesions of indomethacin-treated mice as compared to lesion scores for diluent control-treated mice was seen during most of the treatment period, the differences were only statistically significant toward the latter period of treatment. For these latter time points, the effect sizes (Cohen’s *d*) were moderate to large, ranging from 0.422 on day 90 to 2.15 on day 120. Shown in Figure 2C are scores for each individual mouse at various time points during the course of treatment. By the endpoint of the study (day 139) two mice in the diluent control group had progressed to advanced HNSCC (score = 4), bearing exophytic tumors, whereas no mice in the indomethacin group progressed to this stage during the study (Figure 2C and examples in Figure 2D). Furthermore, 4 of the 5 mice in the diluent control group had lesion scores of 3 or higher at the endpoint of the study, compared to one mouse in the indomethacin group. These data show that administering indomethacin to mice with 4-NQO-induced premalignant lesions slows progression to HNSCC.

### Effect of Administering Indomethacin to Mice with 4-NQO-Induced Premalignant Lesions on Cellularity of Cervical Lymph Nodes

**Figure 3:**
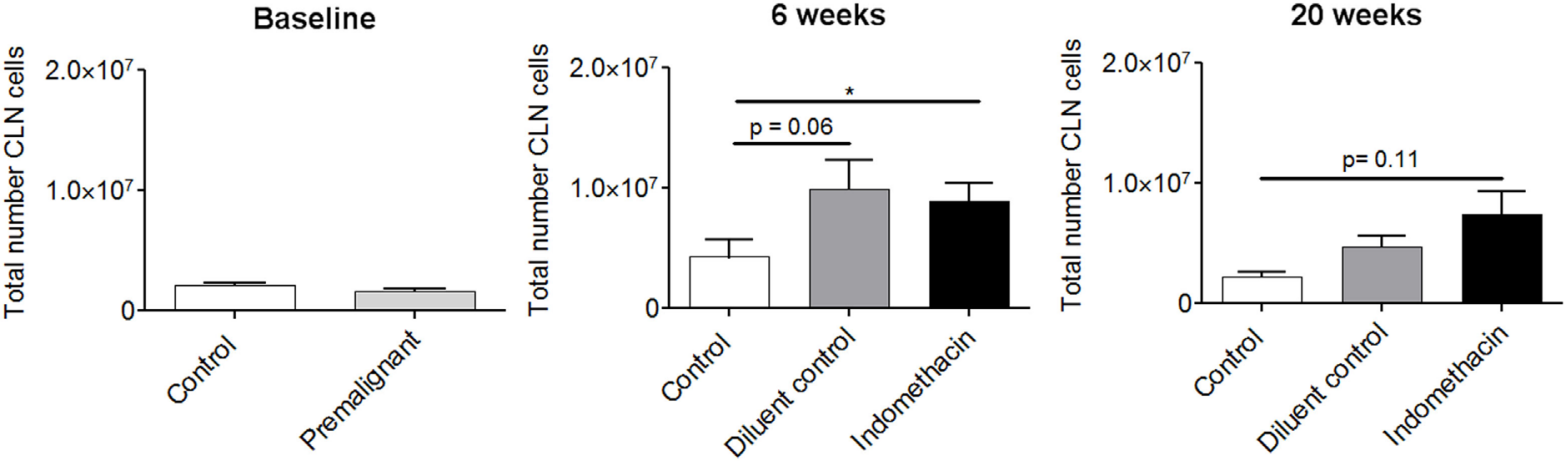
**Effect of administering indomethacin to mice with 4-NQO-induced premalignant lesions on cellularity of cervical lymph nodes**. Cervical lymph nodes (CLN) from mice receiving indomethacin or diluent control treatments were harvested at baseline, 6 and 20 weeks post-initiation of treatment. Lymph nodes were dissociated to single cell suspensions and the number of trypan blue-excluding cells was counted. Results are from five mice per group per time point. Data represent mean ± SEM. *p < 0.05 (two-tailed Student’s *t*-test).

Previous studies had shown that the cervical lymph nodes of mice with 4-NQO-induced HNSCC tumors undergo hyperplasia (24). The current study sought to determine if indomethacin treatment impacted on the total cellularity of cervical lymph nodes, as premalignant lesions progressed to HNSCC. As seen in Figure 3, there were no differences in the total number of cervical lymph node cells in healthy control and premalignant lesion-bearing mice at the onset of indomethacin treatment, when premalignant oral lesions were initially detected (baseline). At 6 weeks of treatment, the number of lymph node cells in premalignant lesion-bearing mice that were treated with either diluent control or indomethacin was increased compared to the number in healthy control mice. The effect size was strong for both control vs. diluent control (1.27) and control vs. indomethacin (1.36). These data suggest that regional lymph nodes exhibit hyperplasia during the progression of premalignant oral lesions toward HNSCC. At the endpoint of the study (20 weeks of treatment), the total number of cervical lymph node cells in indomethacin-treated mice remained increased compared to healthy control mice (Cohen’s *d* = 1.872), whereas the number of lymph node cells in lesion-bearing mice treated with diluent control declined compared to the 6-week time point. The differences in cellularity of lymph nodes from diluent- vs. indomethacin-treated mice did not reach statistical significance, but these data nevertheless show increased lymph node cellularity in mice bearing premalignant lesions and a decline in this cellularity as lesions progress to cancer.

### Effect of Administering Indomethacin to Mice with 4-NQO-Induced Premalignant Lesions on the Percentage and Absolute Number of CD8^+^ T cells Expressing IFN-γ in the Cervical Lymph Nodes

**Figure 4:**
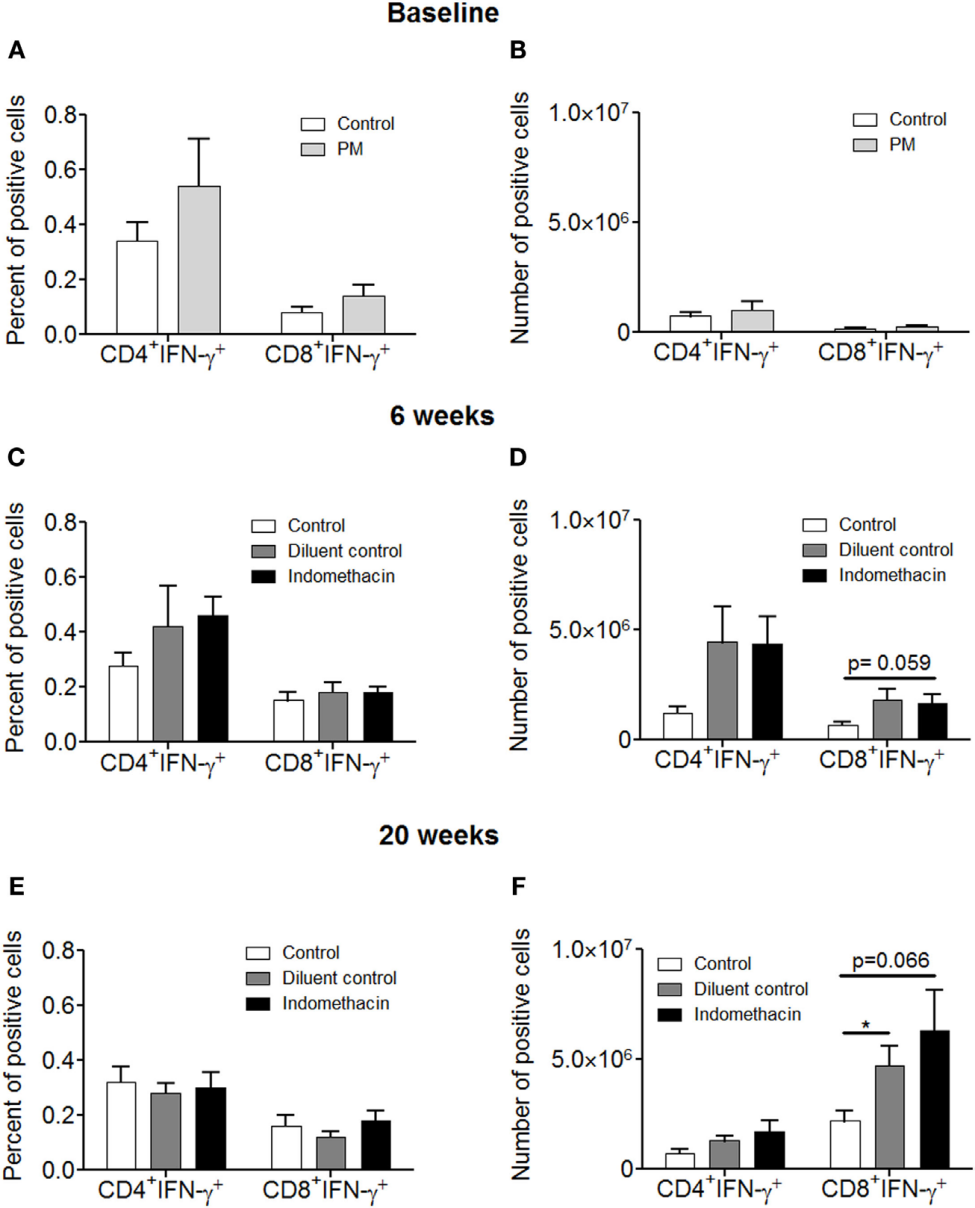
**Administering indomethacin to mice with 4-NQO-induced premalignant lesions increases the number of IFN-γ-expressing CD8^+^ T cells in cervical lymph nodes**. Graphical representation of the percentage and the absolute number of IFN-γ-expressing cervical lymph node cells of healthy control mice and premalignant lesion-bearing mice at baseline **(A,B)** and of premalignant lesion-bearing mice receiving indomethacin or diluent control treatment for 6 **(C,D)** and 20 weeks **(E,F)**. Five mice were examined for each group per time point. The absolute number of positive-staining cells was calculated by multiplying percent positive cells by the number of cervical lymph node cells for each mouse. Data represent mean ± SEM. *p < 0.05 (two-tailed Student’s *t*-test).

Our studies described above (Figure 1) showing that inhibiting prostaglandin production by premalignant lesion cells leads to their induction of T cell cytokine production prompted studies to determine the impact of indomethacin treatment of premalignant lesion-bearing mice on the production of IFN-γ by their lymph node cells. Indomethacin or diluent control was administered to premalignant lesion-bearing mice. Cervical lymph node cells were then analyzed for both the frequency and absolute numbers of T cells expressing IFN-γ at 6 and 20 weeks post-initiation of treatment (Figure 4). At baseline, there was a trend (not significant) toward increased percentages of CD4^+^ and CD8^+^ T cells expressing IFN-γ (Figure 4A), but no difference in absolute numbers of these cells (Figure 4B) in the cervical lymph nodes of premalignant lesion-bearing mice compared to the lymph nodes of healthy control mice. At 6 weeks after onset of treatment, the percentage (Figure 4C) and, more prominently, the absolute number (Figure 4D) of CD4^+^ and CD8^+^ T cells expressing IFN-γ was increased in premalignant lesion-bearing mice, regardless of whether they were treated with control diluent or indomethacin. The effect size was large for both control vs. diluent control (Cohen’s *d* = 1.30) and control vs. indomethacin (Cohen’s *d* = 1.59). However, at 20 weeks, the percentages of CD4^+^ and CD8^+^ cells expressing IFN-γ in lymph node cells of lesion-bearing mice declined (Figure 4E). However, there was an increase in the absolute number of CD8^+^ cells expressing IFN-γ, which was more prominent for indomethacin-treated mice (Figure 4F, Cohen’s *d* = 1.34 for control vs. indomethacin). These results indicate a progressive increase in the number of IFN-γ-expressing CD8^+^ lymph node cells in mice bearing premalignant lesions from baseline to 6 and 20 weeks of indomethacin treatment.

### Effect of Administering Indomethacin to Mice with 4-NQO-Induced Premalignant Lesions on Their Spleen Cell Cytokine Production

**Figure 5:**
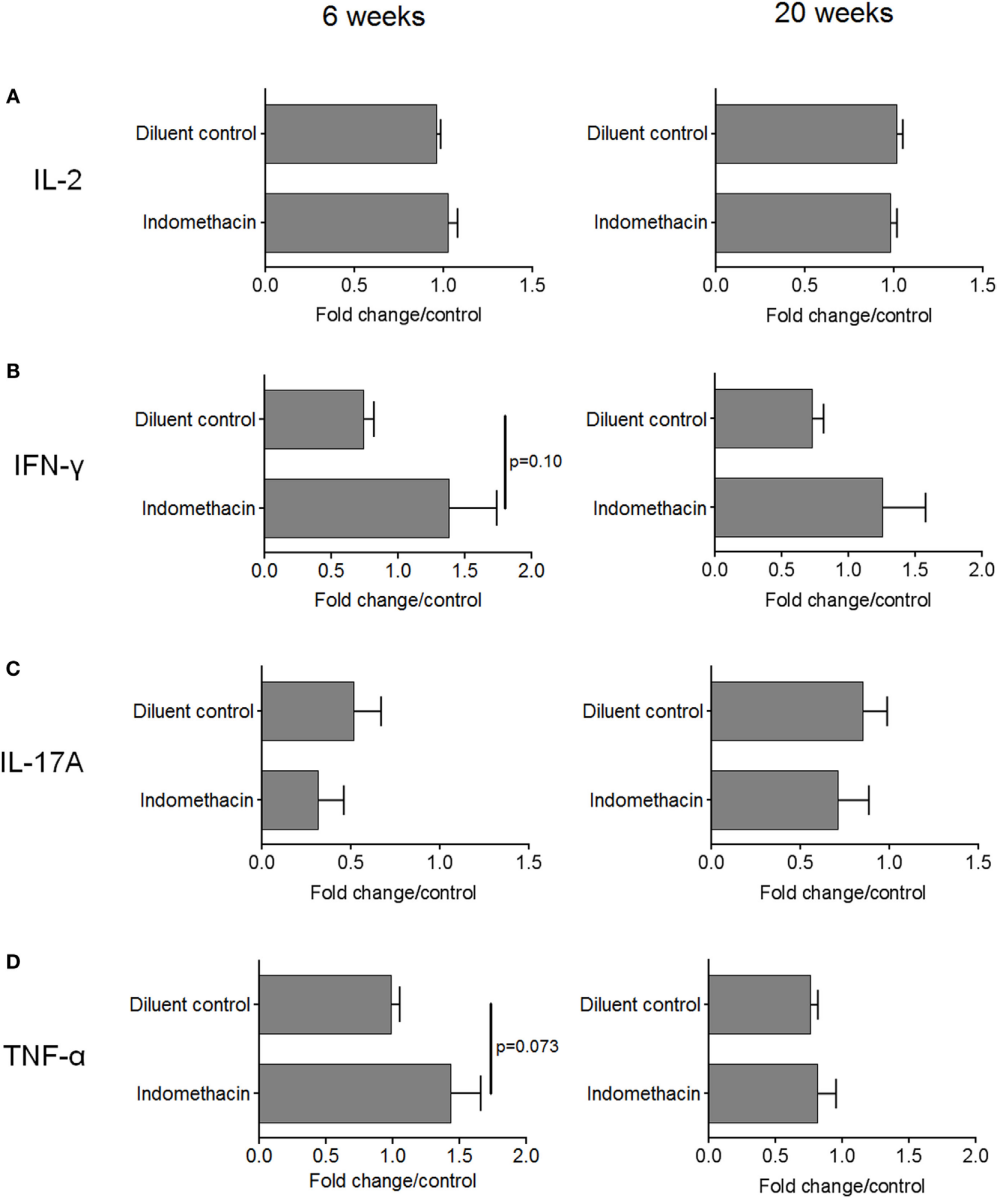
**Administering indomethacin to mice with 4-NQO-induced premalignant lesions increases spleen cell cytokine production at 6 and 20 weeks post-onset of treatment**. Spleens from mice receiving indomethacin or diluent control treatments were collected at 6 and 20 weeks after onset of diluent control or indomethacin treatment. Spleen cells were cultured for 72 h at 1 × 10^6^ cells/well and supernatants were collected for quantitation of secreted IL-2 **(A)**, IFN-γ **(B)**, IL-17A **(C)**, and TNF-α **(D)**. Five mice were examined for each group per time point. Spleen cells from untreated healthy control C57BL/6 mice served as controls. Data are shown as fold change from values for spleen cells from healthy control mice. Data represent mean ± SEM (two-tailed Student’s *t*-test).

While the above studies assessed the impact of treating premalignant lesion-bearing mice with indomethacin on regional lymph node cell expression of IFN-γ, studies were conducted to determine if there was a systemic effect on cytokine production. This was accomplished by measuring levels of cytokines secreted by spleen cells of premalignant lesion-bearing mice after 6 and 20 weeks of diluent or indomethacin treatment. Whereas, spleen cells from diluent control-treated lesion-bearing mice produced lower levels of IFN-γ compared to spleen cells from healthy control mice at the endpoint of the study, treatment with indomethacin enhanced their production of IFN-γ (Table 2). Analysis of the systemic effects of COX inhibition was expanded to include earlier time points of indomethacin treatment (Figures 5A–D, fold change/control = 1). Production of IL-2 by spleen cells of mice bearing premalignant oral lesions was similar to that by spleen cells of healthy control mice. This production of IL-2 by spleen cells of mice with premalignant oral lesions was not significantly affected by treating the mice for either 6 or 20 weeks with indomethacin or diluent control (Figure 5A). In contrast, the level of IFN-γ produced by spleen cells of diluent-treated mice bearing premalignant oral lesions was reduced at both 6 and 20 weeks compared to levels of IFN-γ produced by spleen cells of healthy control mice (Figure 5B). Treating lesion-bearing mice with indomethacin increased their spleen cell production of IFN-γ compared to the amount produced by spleen cells from diluent control-treated mice or from healthy control mice at both 6 and 20 weeks post-initiation of treatment. The effect size for the level of IFN–γ in diluent control vs. indomethacin was moderately strong (*d* = 0.774 at 6 weeks, *d* = 0.540 at 20 weeks). The level of IL-17A produced by spleen cells of either diluent- or indomethacin-treated mice bearing premalignant lesions was reduced compared to levels produced by healthy control mice, especially at the 6-week time point (Figure 5C). Although not statistically significant, IL-17A production by spleen cells of mice receiving indomethacin tended to be further decreased compared to the level produced by spleen cells of mice receiving diluent control treatment (Cohen’s *d* = 0.429 at 6 weeks). Premalignant lesion-bearing mice that were treated with diluent control produced similar levels of TNF-α as did spleen cells of healthy control mice (Figure 5D). Indomethacin treatment increased TNF-α production by spleen cells of premalignant lesion-bearing mice at 6 weeks post-onset of treatment, although this difference was not evident 20 weeks post-onset of treatment. Overall, these data indicate that presence of premalignant oral lesions can alter cytokine production not only in regional lymph node cells, but also in the spleen. They also show that administering indomethacin to mice with 4-NQO-induced oral lesions can enhance production of select Th1/inflammatory mediators.

**Table 2:** **Impact of administering indomethacin to mice with 4-NQO-induced premalignant lesions on spleen cell cytokine production**.

Cytokine (pg/ml)	Control	4-NQO + diluent control	4-NQO + indomethacin
IL-2	120.1 ± 14.7	122.0 ± 4.1	117.9 ± 4.2
		*p* = 0.4945	
IFN-γ	57.2 ± 8.7	41.5 ± 5.0	71.9 ± 18.3
		*p* = 0.1271	
IL-17A	7.9 ± 1.2	6.8 ± 1.1	5.1 ± 1.4
		*p* = 0.3369	
TNF-α	27.1 ± 3.1	20.7 ± 1.4	22.0 ± 3.8
		*p* = 0.7471	

*Spleens from mice receiving indomethacin or diluent control treatments were collected at study endpoint (20 weeks post-initiation of treatment). Spleen cells were cultured for 72 h at 1 × 10^6^ cells/well and supernatants were collected for quantitation of secreted IL-2, IFN-γ, IL-17A, and TNF-α. Five mice were examined for each group. Spleen cells from untreated healthy control C57BL/6 mice served as controls. Data are presented as pg/ml and represent mean ± SEM (two-tailed Student’s *t*-test)*.

## Discussion

Patients with established HNSCC tumors exhibit pronounced immune suppression (2–4). COX-2 expression is upregulated in HNSCC tumors and a few studies have pointed toward PGE_2_ as a contributor to the immunosuppressive environment of HNSCC (4, 11–14). COX-2 inhibitors are being explored as possible adjuvants to traditional therapies for HNSCC patients. In a few clinical trials, administration of celecoxib as an adjuvant therapy has been associated with significant response rates (22, 31, 32). One retrospective study looking at the relationship between the use of non-selective cyclooxygenase inhibitors and overall survival in HNSCC patients found that median survival was increased among COX inhibitor users, although the regimen/type of COX inhibitor use was not controlled for in this study (21). A critical gap remains in our knowledge of how COX inhibition impacts on the T cell responses in HNSCC patients. Furthermore, the role of PGE_2_ in the premalignant lesion environment has not been previously defined. Our studies suggest that PGE_2_ may be playing an immune modulatory role before HNSCC tumors are established, and that there may be a critical window during the premalignant lesion stage in which COX inhibitors can be administered to facilitate cytokine responses and slow progression to tumor.

Previously, our lab had shown that premalignant lesion cells established from the 4-NQO murine model of oral carcinogenesis secrete increased levels of several immune modulating factors, including PGE_2_, compared to HNSCC cells (27). The current study shows that treatment of premalignant lesion cells with indomethacin, a pan-COX inhibitor, increased their induction of spleen cell production of Th1-associated cytokines. In contrast, treatment of HNSCC cells with indomethacin did not increase their induction of spleen cell Th1-type cytokines, suggesting that it may be more feasible for PGE_2_ inhibitors to stimulate Th1-type immune responses in the premalignant lesion environment. Inhibiting prostaglandin production by premalignant lesion cells also led to their induction of spleen cell production of IL-10, so the effect of indomethacin treatment cannot be classified singularly as “pro-Th1.” The concurrent increase in IL-10 may be explained as a consequence of an initial increase in Th1-type cytokines, as a mechanism to “turn off” the inflammatory response, or PGE_2_ may be altering the production of both Th1-type and Th2-type cytokines in the premalignant lesion environment. To further delve into the mechanism by which COX inhibition impacts on T cell activity, future studies should include phenotypic analysis of CD4^+^ and CD8^+^ T cells by flow cytometry.

PGE_2_ has been shown to play an immunosuppressive role by multiple means. In the tumor environment, PGE_2_ has been shown to directly suppress macrophage function and inhibit IL-12 secretion by dendritic cells, resulting in a Th2-skewing of mature helper T cells (33–37). Furthermore, PGE_2_ has been shown to directly inhibit IL-2 and IFN-γ production by antigen-specific T cells (38, 39). Aside from direct immune inhibition, PGE_2_ also functions to recruit and sustain a population of MDSCs in the tumor environment, which secrete immunosuppressive molecules and also function to inhibit the T cell response (40–42). In a model of lung carcinoma, inhibiting PGE_2_ production led to a shift in cytokine production by T cells toward a more Th1-type response and decreased tumor burden (23). Because earlier studies in our lab had shown that significant immune changes take place at the premalignant lesion stage (24, 27, 43), the current study sought to determine how inhibiting PGE_2_ production at this stage impacts on T cell reactivity and progression to HNSCC. Administration of indomethacin, a pan-COX inhibitor, to mice with 4-NQO-induced premalignant lesions slowed progression of lesions to cancer. To analyze how indomethacin treatment impacts on the local immune response, T cells from cervical lymph nodes of indomethacin- vs. diluent control-treated mice were analyzed for IFN-γ expression. Mice receiving indomethacin treatment had an increase in the number of CD8^+^IFN-γ^+^ T cells in the cervical lymph nodes, which became more prominent with a more prolonged period of indomethacin treatment. These data suggest that COX inhibition at the premalignant lesion stage sustains the activation of tumor-localized T cells and may be a mechanism by which administration of indomethacin resulted in a significantly improved clinical response. Administration of indomethacin at the premalignant lesion stage also impacted on the systemic immune response, although to a lesser extent. Spleen cells from indomethacin-treated mice secreted increased levels of IFN-γ and TNF-α compared to spleen cells from control-treated mice, at 6 weeks post-treatment, suggesting that indomethacin also induces a systemic Th1-type response. At 20 weeks post-treatment, spleen cell IFN-γ secretion remained higher in the indomethacin-treated group, suggesting that, as observed in the cervical lymph nodes, a more prolonged period of indomethacin treatment is associated with an activated Th1-type response. Overall, the data show that administration of indomethacin to mice with 4-NQO-induced lesions significantly improves clinical outcome and results in sustained T cell cytokine production in regional lymph nodes and spleen.

Future studies with larger cohorts of mice will be necessary to investigate the role of PGE_2_ in modulating T cell activity as premalignant lesions progress to tumor. Flow cytometric analysis of T cells in the cervical lymph nodes of indomethacin-treated vs. diluent control-treated lesion-bearing mice should be expanded in the future to include Th2-associated IL-4 and IL-10, Th17-associated IL-17A and CD8^+^ T cell-associated granzyme B to further delineate the impact of prostaglandins on T cell cytokine and protease production in mice with premalignant oral lesions. Proliferation in responses to lesion or tumor antigens by isolated CD4^+^ and CD8^+^ T cells from the cervical lymph nodes of indomethacin-treated vs. diluent control-treated mice would also provide insight into the mechanism of how inhibiting PGE_2_ production at the premalignant lesion stage impacts on the anti-lesion or antitumor response.

The origination of oral cancer often can be detected as leukoplakias or erythroplakias, commonly developing on the tongue and floor of mouth. Studies in the 4-NQO model of oral carcinogenesis show that premalignant lesion cells themselves produce mediators, including PGE_2_, which may be modulating the immune response well before HNSCC is established. The current study suggests that, inhibiting PGE_2_ production at the premalignant lesion stage may have the capacity to sustain a Th1-type immune response and to slow progression to tumor. Importantly, the current study suggests that, administration of immunotherapies, such as COX-2 inhibitors, to patients with premalignant lesions may boost the antitumor response and improve clinical outcomes.

## Author Contributions

SJ and MY equally participated in the planning and design of the studies. SJ conducted the laboratory analyses and MY participated in the data analysis. Both authors contributed to the preparation of the manuscript, and have read and approved the manuscript for submission.

## Conflict of Interest Statement

The authors declare that the research was conducted in the absence of any commercial or financial relationships that could be construed as a potential conflict of interest.
